# Revolutionary NIR-activated silicon nanoparticles: precision-controlled release and targeted 3D cancer cell destruction

**DOI:** 10.1039/d4ra08889a

**Published:** 2025-02-14

**Authors:** Vy Anh Tran, Nguyen Huy Hung, Thu Thao Thi Vo, Seong Soo A. An, Sang-Wha Lee, Hun Jeong, Mario A. Tan

**Affiliations:** a Deparment of Material Science, Institute of Applied Technology and Sustainable Development, Nguyen Tat Thanh University Ho Chi Minh City 700000 Vietnam tavy@ntt.edu.vn; b Center for Advanced Chemistry, Institute of Research and Development, Duy Tan University 03 Quang Trung Da Nang 550000 Vietnam; c Faculty of Natural Sciences, Duy Tan University 03 Quang Trung Da Nang 550000 Vietnam; d Department of Food Science and Biotechnology, Gachon University 1342 Seongnamdaero, Sujeong-gu Seongnam-si 13120 Republic of Korea; e Department of Bionano Technology, Bionano Research Institute, Gachon University Seongnam-si 1342 Gyeonggi-do 461-701 Republic of Korea; f Department of Chemical and Biological Engineering, Gachon University Seongnam-si 1342 Gyeonggi-do 461-701 Republic of Korea; g Department of Natural Products & Biotechnology, Jeonbuk Science College Jeongeup 56204 Republic of Korea; h College of Science and Research Center for the Natural and Applied Sciences, University of Santo Tomas Manila 1015 Philippines matan@ust.edu.ph

## Abstract

In cancer therapy, controlled and targeted drug release systems are essential to maximize therapeutic outcomes while minimizing adverse effects. This study introduces an innovative mesoporous silicon nanoparticle (MSN) platform, functionalized with the natural anticancer agent dieckol (Di) and designed for precise drug delivery activated by near-infrared (NIR) irradiation. By embedding Di and grafting fluorescent organic conjugates onto the MSN surface, this innovative nanocarrier demonstrates exceptional sensitivity to NIR stimuli and potent chemo-photothermal effects. Notably, drug release remains stable across different pH conditions (7.4, 6.5, and 5.5), ensuring consistent therapeutic delivery. However, upon NIR exposure, the release can be selectively accelerated, enabling precise, real-time, and on-demand drug release control for enhanced treatment efficacy. Cytotoxicity tests revealed that IPSi-Dox-Di-DQA nanoparticles exhibited potent dose-dependent inhibition of cancer cell growth (SH-SY5Y and B16-F10), while sparing healthy cells (HEK-293), highlighting their specificity. Furthermore, advanced 3D cell viability assays mimic the complexities of *in vivo* cancer environments, with spheroid disintegration under nanoparticle treatment underscoring the platform's powerful anticancer potential. These findings position IPSi-Dox-Di-DQA nanoparticles as a promising frontier in the development of selective, effective cancer therapeutics through synergistic NIR-controlled drug release and mitochondrial targeting.

## Abstract 2

This study develops NIR-activated mesoporous silicon NPs for targeted cancer therapy. Functionalized with dieckol and dequalinium, they enable pH-sensitive drug release and enhance chemo-photothermal effects, selectively eliminating cancer cells.

## Graphical abstract

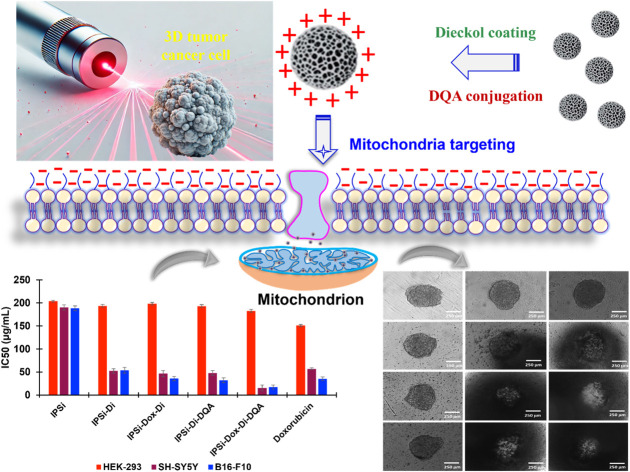

## 1 Introduction

A smart drug delivery system (DDS) is one of the most promising therapeutic strategies for effective cancer therapy. Importantly, a DDS should not only exhibit high therapeutic efficacy but also be biocompatible, degradable, and safe for clinical use.^1–3^ DDS should have the capability of controlled drug release which can provide the minimum therapeutic concentration in the target area without developing drug-resistant cancer cells.^4,5^ In this aspect, stimuli-induced drug release can be the best alternative to conventional DDSs, as they are accurately controlled by near-infrared (NIR) irradiation, pH conditions, exposure duration,^6^ and composition.^7–12^ Multiple integrations of different functional techniques are highly necessary to improve anti-cancer efficacy.^13–17^ To produce potential anticancer effects on cancer treatment, mitochondria-targeted DDS should have imaging capabilities, therapeutic action, and selectivity in targeting cancer cell organelles at the same time.^18–20^

Mesoporous silicon nanoparticles (PSi) are a highly promising class of nanomaterials, characterized by their unique structural and chemical properties, making them highly attractive for biomedical applications, particularly in the field of cancer therapeutics.^21,22^ These nanoparticles feature a well-defined porous structure with an interconnected channel network and a high surface area, allowing efficient drug loading and delivery.^12^ The mesoporous structure of these silicon nanoparticles provides an ideal platform for the controlled release of therapeutic agents, enabling precise modulation of drug delivery kinetics to maximize therapeutic efficacy while minimizing side effects.^7,8,12^

A fascinating aspect of mesoporous silicon nanoparticles is their significant potential in the realm of anticancer therapy. The tailored pore size and surface chemistry of MSNPs allow for the encapsulation and targeted delivery of a wide range of anticancer drugs, nucleic acids, or imaging agents.^23^ This capability enables site-specific drug release, prolonged circulation times, and improved cellular uptake, leading to enhanced therapeutic outcomes. Additionally, the biocompatibility and biodegradability of MSNPs contribute to their appeal in cancer treatment, as they offer a safer and more sustainable approach compared to some traditional drug delivery systems.^13,15,19^ Several studies underscore the anticancer potential of mesoporous silicon nanoparticles. MSNPs were employed for the targeted delivery of doxorubicin, demonstrating enhanced drug accumulation in cancer cells and improved therapeutic outcomes.^24^ The biocompatibility and degradability of MSNPs contribute to their appeal in cancer therapeutics, providing a safer and more sustainable alternative to conventional drug delivery systems.^25^ These nanomaterials hold great promise in revolutionizing cancer treatment strategies, offering a platform for precision medicine with reduced systemic toxicity and improved patient outcomes.^26^

Aberrations in mitochondrial physiology are linked to various diseases, as mitochondria are double-membrane-bound organelles responsible for ATP production, regulation of intracellular calcium homeostasis, reactive oxygen species generation, intrinsic apoptotic pathway activation, and hormone synthesis.^27–29^ Despite abundant oxygen availability, cancer cells predominantly rely on high-rate glycolysis for energy production. Consequently, mitochondria are increasingly recognized as a key target for therapeutic intervention.^30^ The inner mitochondrial membrane is highly folded and compartmentalized, making it challenging for pharmacological molecules to penetrate the mitochondria.^31^ Compared to the plasma membrane, the potential of the mitochondrial membrane in cancer cells is three to five times higher, indicating a strongly negative state. As a result, the mitochondria organelle readily accumulates positively charged molecules.^32^ Utilizing particular compounds like triphenylphosphonium (TPP), dequalinium (DQA), mitochondria penetration peptides (MPPs), or mitochondria targeting signal peptides, a number of viable mitochondrial delivery systems have been developed.^33,34^ Additionally, employing graphene oxide, mitochondria-targeting nanoparticles have been developed,^35^ lipid-coated carbon-quantum dots, silica NPs, and gold NPs to deliver the therapeutic agent to mitochondria without drug resistance.^36–40^ Conjugate-loaded NPs were shown to localize in mitochondria where they caused damage to the mitochondrial membrane, increasing cytotoxicity and ultimately leading to apoptotic cell death.^32^

**Fig. 1:**
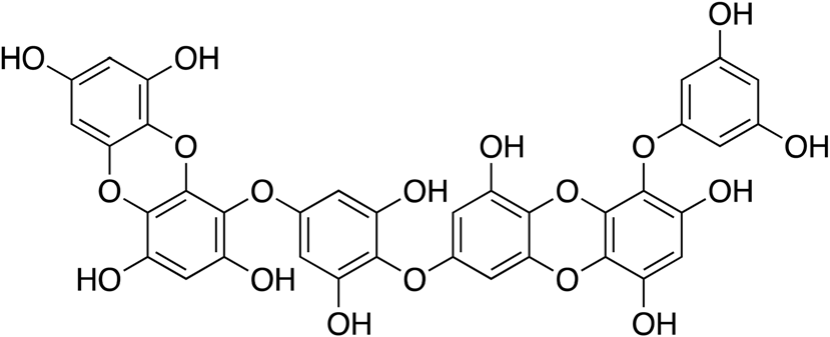
Structure of dieckol.

Dieckol (Di) (Fig. 1) is a natural polyphenol compound (so-called phlorotannins) that has low aqueous solubility because of multiple hydrophobic aromatic rings despite several hydroxyl groups.^41^ The phlorotannin components isolated from several brown algae of genus *Eisenia* and *Ecklonia* possess diverse biological effects, including anti-inflammatory and antioxidative, antiviral, and anticancer activities.^42^ Besides, the intrinsic stability of the phenolic Di structure can lower the possibility of early release of therapeutic drugs from mesoporous nanocarriers.^43^ In this context, Di conjugations with the PSi NPs system can provide strong anticancer efficacy, chemo-photothermal sensitivity, and stimuli-responsive controlled drug release.^32,44,45^ Also, polar hydroxyl and polyaromatic groups of Di affect hydrogen bonding, π–π stacking interactions, and self-assembly behavior.^46,47^

In this study, well-defined mesoporous silicon NPs (PSi) were first functionalized with fluorescent organic conjugates, yielding fluorescent PSi (IPSi). The IPSi was then coated with an anticancer Di interlayer, which exhibited a consistent drug release profile across varying pH (pH 7.4 to pH 5.5), while NIR irradiation induced the highest release rate at pH 7.4. Further conjugation with DQA endowed the nanocarrier with potent *in vitro* antiproliferative effects. To further evaluate its biological activity, we also investigated the *in vitro* 2D and 3D cytotoxicity against a normal cell line (human embryonic kidney HEK-293) and two cancer cell lines, human neuroblastoma (SH-SY5Y) and murine melanoma tumor (B16-F10).

## 2 Materials and methods

### 2.1 General considerations

Tetraethyl orthosilicate (TEOS, 99%), 3-aminopropyltrimethoxysilane (APTMS, 97%), cetyltrimethylammonium bromide (CTAB, 99%), ammonium fluoride (NH_4_F, 99.99%), magnesium (Mg, 20-230 mesh, 98%), fluorescein isothiocyanate (FITC, 90%), doxorubicin hydrochloride, dimethyl sulfoxide (DMSO, ≥99.9%), and dequalinium (DQA, ≥95%) were purchased from Sigma-Aldrich Company. Without additional filtration, ethanol, and water (HPLC grade) were used exactly as supplied. Di (99%) extracted from seaweed. The remaining chemicals were utilized exactly as supplied and were of the best quality commercially available. Glassware was cleaned using an acidic solution of HNO_3_ : HCl (1 : 3 vol/vol%) and then rinsed several times with deionized (DI) water.

### 2.2 Synthesis and modification of nanomaterials

#### 2.2.1 Silica nanoparticles

Through a sol–gel reaction with TEOS, the cationic surfactant CTAB was used to create silica nanoparticles. Briefly, 200 mL of water, 0.50 g of CTAB, and 1.0 g of NH_4_F were combined and heated to 75 °C while being vigorously stirred. After the components were fully dissolved, 4.5 mL of TEOS was added dropwise and stirred for 6 h at 75 °C to produce a milky white solution. The milky solution was centrifuged, repeatedly rinsed with water and ethanol, then dried in a freeze dryer to get the white solid of silica nanoparticles. After dispersing the white precipitates in 150 mL of ethanol with 2.0 mL of conc. HCl, the mixture was refluxed at 70 °C for 12 h to eliminate the CTAB (surfactant template). To make sure the surfactant was completely removed, this process was performed again. The final silica nanoparticle products underwent centrifugation, several water washes, and a 12 hour freeze-drying period.

#### 2.2.2 Conversion to mesoporous silicon NPs from silica nanoparticles

Six hundred (600) mg of Mg powder (325 mesh) and 700 mg of MSN were combined and added to the reactor in the glove box that was filled with argon environment. In an argon environment, the reactor was heated to 700 °C for six h (5 °C min^−1^) in a tube furnace before being cooled to ambient temperature. To get rid of MgO, Mg_2_Si, and unwanted products, the goods were submerged in 40 mL of HCl (2 M) for seven hours. This was followed by several water washes. To get rid of any remaining silica, large volumes of a 7 wt% HF solution were used to wash the items. The powder was spread in ethanol, washed with water several times, and then freeze-dried for 24 hours.

#### 2.2.3 Doxorubicin loading, dieckol coating, and QDA conjugation

##### 2.2.3.1 Doxorubicin loading

A 300 mg of mesoporous silicon was dissolved in 15 mL of water with 100 mg of doxorubicin hydrochloride in order to load the medication. To possibly load most of the doxorubicin, the combined solution was agitated for 12 h. Centrifugation was used for 10 min at 6000 rpm to separate the mesoporous silicon loaded with doxorubicin. By calculating the difference between the starting and residual concentrations of doxorubicin in the solution, the loading quantity of doxorubicin was calculated, and the supernatant was collected.

##### 2.2.3.2 Coating with Di layer

After multiple interactions with the OH and O-groups of mesoporous silicon, certain entities such as polyphenols and COOH groups covered the Di layer on the mesoporous silicon surface. Despite the presence of numerous polyphenol groups, Di exhibited considerable solubility in the aqueous phase, facilitating the straightforward separation of the supernatant from the sediment at the bottom. The mesoporous silicon was then combined with the supernatant containing dissolved Di, and the coating procedure was conducted under light-free conditions at RT. Subsequently, the Di-coated mesoporous silicon underwent centrifugation and multiple washes with ample water to eliminate any remaining contaminants.

##### 2.2.3.3 Conjugation with DQA

DQA-conjugated mesoporous silicon was created by the interplay between Di's polyphenol groups and DQA's amine groups. 6 mL of a mesoporous silicon solution was mixed with 10 mg of DQA. The interaction mixture was gently agitated using a magnetic stirrer for 20 min at RT. To get rid of any remaining contaminants, the finished product was centrifuged and then rinsed with water.

### 2.3 NIR irradiation and controlled drug release

At 35 ± 1 °C, the mesoporous silicon samples were continuously stirred while being dissolved in 15 mL of PBS. The moment the mesoporous silicon samples were put into the PBS solution, the *in vitro* drug release assay began. By repeatedly sampling the solution, UV-vis spectroscopy was employed to assess changes in the absorbance of the released doxorubicin in the PBS at 496 nm. The standard curve, which was based on the linear relationship between absorbance and corresponding concentration, was used to translate the absorbance values into the quantities of doxorubicin that were released.

Mesoporous silicon samples were used in in vitro release assays in 15 mL of PBS solutions at three pHs of pH 7.4, pH 6.5, and pH 5.5 to examine the pH-sensitive drug release behavior of the samples. Periodically, the NIR irradiation (808 nm, 1.0 W cm^−2^) was used for 5 min of exposure at release periods of 0, 5, 10, 20, and 30 hours, respectively. The solution was routinely collected throughout the continuous release procedure, and the absorbance was determined using UV-vis spectroscopy at 301 nm. Ultimately, the fractional release of the drug was plotted against corresponding times, and the released quantities of the drug were computed using the linear correlation of the standard curve.

### 2.4 *In vitro* antiproliferative activity

#### 2.4.1 Cell culture

Human embryonic kidney HEK-293 (CRL-1573), human neuroblastoma SH-SY5Y (CRL 2266), the murine melanoma tumor B16-F10 (CRL 6475) were obtained from ATCC (Manassas, VA, USA), and grown at 37 °C in a moistened 5% CO_2_ incubator in Dulbecco's modified Eagle's medium (DMEM) supplemented with 10% fetal bovine serum (FBS) and 1% antibiotics (streptomycin/penicillin, 1000 U mL^−1^).

#### 2.4.2 2D cytotoxicity assay

A mitochondrial-based cell viability assay, ATP luminescence, was used to measure cytotoxicity.^48^ Cells (1 × 10^4^ cells per well) were seeded in 96-well plates and incubated for 24 h. Cells were then exposed to the nanoparticles (0–100 μg mL^−1^) for 48 h. After treatment, cells were washed three times with PBS, added with fresh 100 μL media, and incubated for 30 min before adding 100 μL of CellTiter-Glo® Luminescent (Promega) reagent. A PerkinElmer Victor-3® multi-plate reader was used to read the luminescence signal after 10 minutes of gentle shaking. Following data analysis, the % cell viability was expressed concerning the control cells (no treatment). Cell viability of doxorubicin (positive control) was also determined at 1–50 μg mL^−1^ concentration ranges.

#### 2.4.3 3D cell viability assay

The 3D cytotoxicity was done following a previous protocol.^49^ Cells (2 × 10^4^ cells per well) were seeded in a 96-well round-bottomed plate, centrifuged at 1000 rpm for 10 min, and incubated for 5 days at 37 °C in a moisturized 5% CO_2_ incubator. The formed spheroidal tumor cells were treated with the nanoparticle IPSi-Dox-Di-DQA. The cell morphology was monitored after 1 h, 24 h, and 48 h at 10× magnification using a JuLi Stage cell imaging system (NanoEntek, Republic of Korea).

### 2.5 Statistical analysis

Data were reported as mean values with standard deviations (mean ± SD). One-way ANOVA was used to examine statistical significance followed by Tukey's HSD test and *p* < 0.05 was considered statistically significant. Using the online resource tool, half-maximal inhibitory doses (IC_50_) were calculated. Using the online resource tool, half-maximal inhibitory doses (IC_50_) were calculated, MLA-“Quest Graph™ IC_50_ Calculator” – AAT Bioquest, Inc., https://www.aatbio.com/tools/ic50-calculator.^50,51^

## 3 Results and discussion

### 3.1 Physico-chemical properties of nanomaterial

**Fig. 2:**
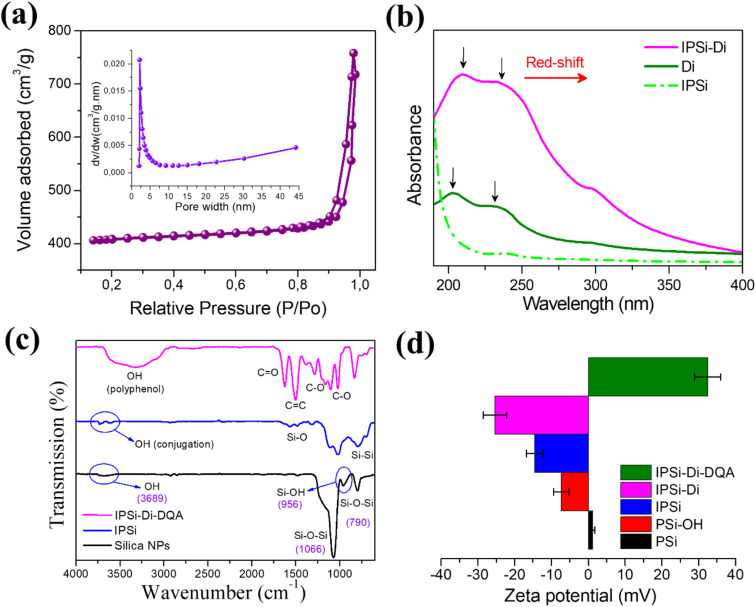
(a) Pore size distribution and nitrogen adsorption/desorption isotherms utilizing the Barrett–Joyner–Halenda (BJH) analysis of IPSI; (b) UV-Vis spectra of IPSi, Di, and IPSi-Di; (c) FTIR spectra of silica NPs, PSi, and IPSi-Di-DQA; (d) zeta potential of PSi, PSi-OH, IPSI, IPSI-Di, IPSI-Di-DQA.

The isotherm curve of mesoporous silicon displayed a hybrid type III/IV isotherm, with a distinctive step-down in the desorption branch associated with the closure of the hysteresis loop, as per the Barrett–Joyner–Halenda (BJH) analysis shown in Fig. 2a. In the pressure range of 0.85–1.0, the pore-filling step of the adsorption isotherm curve was acute, indicating a narrow pore size distribution. The mesoporous silicon exhibited 1407 m^2^ g^−1^ of specific surface area and a pore size distribution of around 2.52 nm. Mesoporous silicon's very porous nature is advantageous for holding vast quantities of anti-cancer medications.

The interaction between Di layers and IPSI as shown by UV-vis spectra is seen in Fig. 2b. The π–π* and n–π* transitions were attributed to the Di absorption peaks that were located at 205 and 236 nm, respectively. Conversely, π–π* and n–π* peaks were seen in Di-coated mesoporous silicon at 211 and 239 nm, respectively. The small redshift of UV-vis absorbance suggests that Di layers interact *via* contact with mesoporous silicon's functional surface groups.

The accompanying FT-IR spectra verified the effective conversion of silica NPs to fluorescent mesoporous silicon and coated Di, DQA (Fig. 2c). It was possible to see the distinctive peaks of silica NPs at 1078 and 791 cm^−1^, respectively, which correspond to the symmetric and antisymmetric stretching vibrations of the Si–O–Si connection in the oxygen-silica tetrahedron. The stretching vibrations of silanol groups and absorbed water were attributed to the weak bands at 3656 and 933 cm^−1^, respectively. The Si–Si bond is identified by a peak at 802 cm^−1^ in the FTIR spectrum of mesoporous silicon, whereas the existence of residual Si–O–Si bonds is indicated by a peak at 1294 cm^−1^. A new wideband developed from 3100 to 3670 cm^−1^ with further coating with the Di layer, and it was identified as the OH groups of polyphenols. Furthermore, Di's C–O vibration was revealed by twin peaks at 1111 cm^−1^ and 1003 cm^−1^.

At each stage of the synthesis of multifunctional PSi, the zeta potential of the result was measured at neutral pH 7.4 to confirm the stepwise surface modification of NPs. After OH conjugation, PSi's zeta potential which had been determined to be +1.20 mV flipped to a negative value of −7.35 mV, signifying the production of hydroxyl groups on the surface of the particle. Because of the APTMS-FITC exposure groups on the surface, the zeta potential of IPSi decreases to a negative value of −14.55 mV after grafting by APTMS-FITC. After the IPSi đoc coating was applied to Di, the zeta potential was found to be −25.34 mV. However, the positively charged ammonium groups of DQA on the surface are what caused the grafting with DQA to shift the zeta potential to a positive value of +32.45 mV (Fig. 2d).

**Fig. 3:**
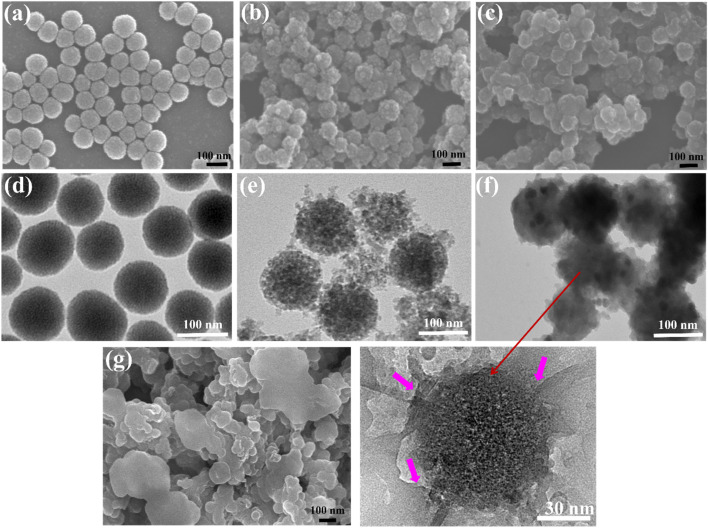
SEM images of (a) silica NPs, (b) PSi, (c) IPSi-Di-DQA, and TEM images of (d) silica NPs, (e) PSi, (f) IPSi-Di-DQA, (g) IPSi-Di-DQA after NIR irradiation and drug release.

The pictures of mesoporous silicon samples at each stage of functionalization, silica NPs, PSi, and IPSi-Di-DQA, obtained using scanning electron microscopy (SEM) and transmission electron microscopy (TEM), are shown in Fig. 3. The silica nanoparticles had a consistent, orbicular structure. The TEM picture amply demonstrated the existence of evenly dispersed mesopores exposed to the outside, with an average particle size of around 105 nm (Fig. 3a and d). These mesopores are abundant, which helps to avoid excessive fusion of reduced Si nanocrystals by enhancing the dissipation of reaction heat and facilitating the passage of magnesium vapor. Due to the HF etching technique' creation of ultrafine Si nanocrystals, several pieces were seen. PSi's spherical form was effectively maintained during the magnesium reduction procedure (Fig. 3b and e). The SEM and TEM images of the Di-coated Psi and DQA conjugation, which produce the smooth surface morphology, are shown in Fig. 3c and f show the very thin layer of Di and DQA layers overlaying PSi in the HR-TEM picture.

### 3.2 *In vitro* drug release by pH control and NIR irradiation

#### 3.2.1 NIR irradiation investigation

**Fig. 4:**
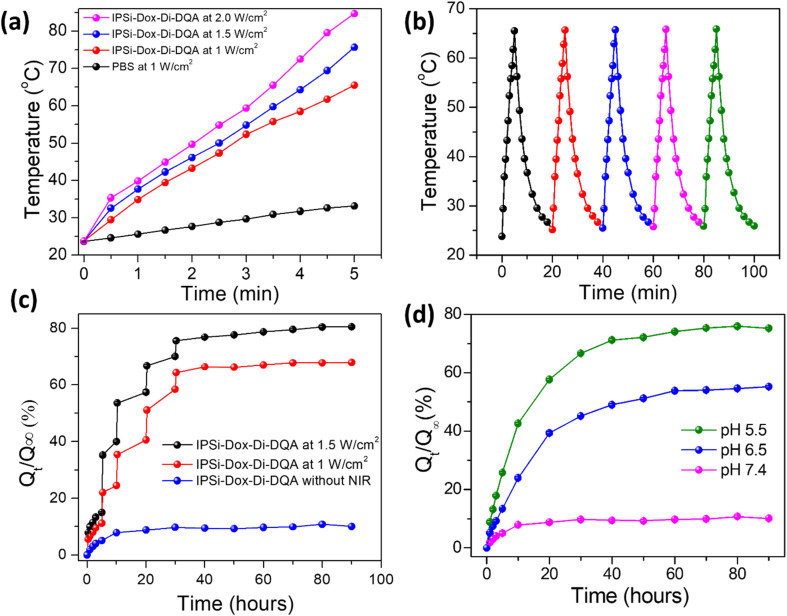
(a) Temperature profiles of PBS (at 1 W cm^−2^), IPSi-Dox-Di-DQA at 1 W cm^−2^, 1.5 W cm^−2^ and 2.0 1 W cm^−2^ with 808 nm laser irradiation for 5 min and then shut off the laser; (b) photothermal conversion cycling test of IPSi-Dox-Di-DQA under five ON/OFF cycles of 808 nm laser irradiation (1 W cm^−2^); (c) cumulative release fraction of Dox from IPSi-Dox-Di-DQA without and with NIR irradiation 808 nm laser, 1.0 W cm^−2^ and 1.5 W cm^−2^ in PBS by periodic irradiations of NIR light for 5 min; (d) doxorubicin release profiles from IPSi-Dox-Di-DQA in PBS at pH 7.4, 6.5, and 5.5.

As-prepared samples were irradiated with NIR light (808 nm, 1.0, 1.5, and 2.0 W cm^−2^) for 5 min, 20 cm of NIR irradiation distance, and 10 J cm^−2^ of energy density in order to examine the photothermal heating effect on silicon nanocarriers. The temperatures of pure PBS rose from 23.8 °C to 33.5 °C under NIR light settings (808 nm, 1.0 W cm^−2^), as shown in Fig. 4a. However, with IPSi-Dox-Di-DQA, using 1.0, 1.5, and 2.0 W cm^−2^, the solution's temperature rose dramatically from 23.8 °C to 65.5 °C, 75.8 °C, and 84.7 °C, respectively. These findings show that because of its high surface area, low reflectivity, and outstanding photothermal conversion efficiency, silicon nanostructure with many mesopores exhibits strong photo-induced hyperthermia (or antireflection). The lines in Fig. 4b show how the temperature of an *in vitro* solution changed after five cycles when it was periodically exposed to NIR light for 20 minutes.

#### 3.2.2 *In vitro* release test with NIR

*In vitro* release studies of IPSi-Dox-Di-DQA were conducted in PBS under NIR irradiation (808 nm, 1.0 W cm^−2^) at pH 7.4 in order to examine the NIR response of drug release behavior. For five minutes, the NIR laser was irradiated intermittently. The solution was routinely sampled during the *in vitro* release procedure, and the absorbance at 496 nm was determined using UV-vis spectroscopy. The release rate of IPSi-Dox-Di-DQA rose dramatically under each NIR light irradiation (808 nm, 1.5 and 1.0 W cm^−2^), as Fig. 4c illustrates.

Following the initial NIR irradiation, the IPSi-Dox-Di-DQA release fraction significantly increased from 15.15% to 35.44% at 1.5 W cm^−2^. Following that, the percentage of release rose to 53.87% (second NIR irradiation at 5 hours), 66.80% (third NIR irradiation at 10 hours), and 69.74% to 76.18% (fourth NIR irradiation at 20 h). At 90 hours, the release fraction asymptotically reached 80.62%. On the other hand, in the absence of NIR irradiation, the release fraction of IPSi-Dox-Di-DQA steadily approached an asymptotic value of 10.54% at 90 hours.

At 1.0 W cm^−2^, the release fraction of IPSi-Dox-Di-DQA slowly reached 22.16% by 1st NIR irradiation from 10.90%, which continuously increased from 24.01% to 34.89% by 2nd NIR irradiation, from 40.43% to 51.28% by 3rd NIR irradiation, and finally reached to 64.40% from 58.29% by the 4th NIR irradiation. After 90 hours, the release fraction progressively got closer to its asymptotic value of 68.0%.

#### 3.2.3 *In vitro* release test by pH changes

At 37 ± 1 °C, the IPSi-Dox-Di-DQA was continuously stirred while being dissolved in 10 mL of PBS. The solution was routinely sampled during the *in vitro* release test in order to use UV-Vis spectroscopy to assess changes in absorbance at 496 nm. Using the standard curve, the computed absorbance changes were converted into released Dox quantities. The cumulative release fraction at 3 hours was 8.94% at pH 6.5 and 17.76% at pH 5.5, as shown in Fig. 4d. Subsequently, at pH 6.5 and pH 5.5, the release fraction increased to 23.90% and 42.7%, respectively, at 10 hours and 45.20% and 66.30%, respectively, at 30 hours. For 50 hours, the dox release process gradually approached an asymptotic value of 51.34% at pH 6.5 and 72.64% at pH 5.

Under low pH conditions, polar hydroxyl and polyaromatic groups of Di affect hydrogen bonding, π–π stacking interactions, and self-assembly behavior. Thus, it leads to breaking the IPSi external coating and leads to a faster Dox release process. However, higher pH (pH 7.4) does not affect this coating much and results in negligible Dox release. Consequently, Fig. 4d illustrates that the IPSi-Dox-Di-DQA revealed a greater variation in release percentages between pH 5.5, pH 6.5, and pH 7.4. Besides controlling the drug release process, chemo-photothermal sensitivity and potent anticancer activity can be obtained using di conjugations with the PSi NPs system.

As presented in Fig. 4c, the cumulative release fraction of Dox from IPSi-Dox-Di-DQA without NIR irradiation demonstrated that the nanoparticles retained most of the drug over an extended period, up to 90 hours, with drug retention capability extending up to 5 days. Regarding the photothermal conversion test of IPSi-Di-DQA, the nanoparticles still exhibited stable thermal conversion, even after 20 ON/OFF cycles under NIR irradiation. SEM images of the nanoparticles after drug release and NIR irradiation showed that their structural integrity remained intact. This confirms the long-term stability of the nanoparticles under NIR irradiation and the drug release process.

**Scheme 1:**
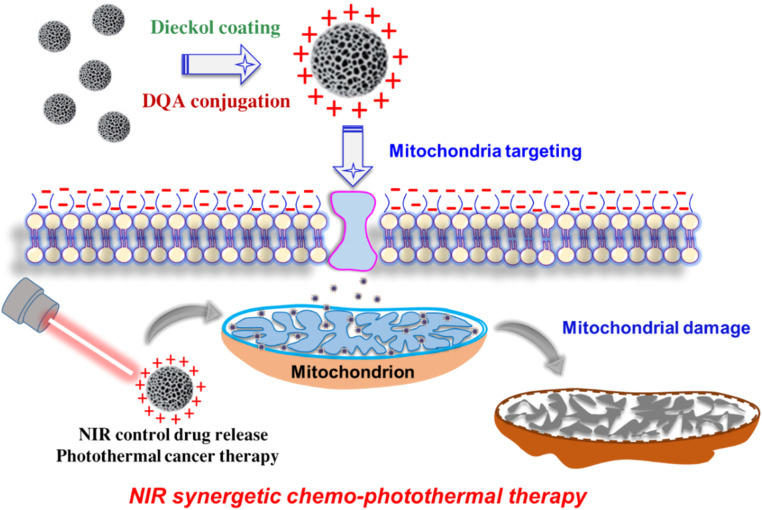
Schematic illustration of IPSi-Dox-Di-DQA, targeted drug delivery to mitochondria of the cancer cell, and NIR synergetic chemo-photothermal therapy.

Because DQA is amphiphilic, it may self-assemble into cationic vesicles that resemble liposomes and take advantage of the very negative Δ*ψ*_m_ seen in cancer cells. That is, by activating nucleases in the inner membrane and matrix of mitochondria, DQA-conjugated NPs may be a useful mitochondriotropic carrier that delivers deadly medications into malignant cells. DQA-conjugated nanoparticles can accumulate specifically in mitochondria through the reduction of membrane potential and damage to the mitochondrial membrane. This ultimately triggers the production of reactive oxygen species (ROS), which can impede the production of ATP and cause apoptosis in cells (Scheme 1). Therefore, it is hypothesized that DQA-conjugated mesoporous silicon has a superior capacity to target malignant cells' mitochondria and provide them with regulated drug release that is sensitive to stimuli.

### 3.3 Cytotoxicity studies on the synthesized nanoparticles

**Fig. 5:**
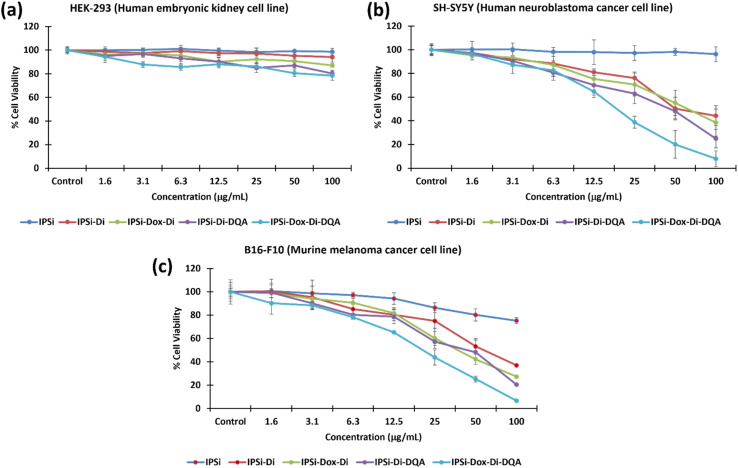
Cell cytotoxicity of the synthesized nanoparticles using the ATP luminescence assay. Normal cells (HEK-293) and cancer cells (SH-SY5Y and B16-F10) were treated with 1.6–100 μg mL^−1^ nanoparticles. The “Control” refers to untreated cells.

Assessment of the cytotoxic potential of the synthesized nanoparticles was performed on HEK-293 human embryonic kidney normal cells, SH-SY5Y human neuroblastoma cancer cells, and B16-F10 murine melanoma cancer cells. ATP luminescence assay was used utilizing 1.6–100 μg mL^−1^ concentrations of the nanoparticles. As shown in Fig. 5, a dose-dependent inhibition of the cancer cells was observed for the nanoparticles apart from the nanoparticle carrier IPSi. Fig. 5a reports the percentage of cell viability, hence, the lower the percentage, the higher the number of cell growth inhibited. None of the nanoparticles showed significant cell growth inhibition using the normal cell line HEK-293 (cell viability > 80%).

A key in Fig. 5b and c is that SH-SY5Y neuroblastoma and B16-F10 melanoma cells exhibited greater sensitivity to the nanoparticles, implying potential applicability in targeted cancer therapy. This finding highlights the importance of further mechanistic studies to elucidate the pathways through which the nanoparticles exert their cytotoxic effects. Additionally, the dose-dependent cytotoxicity indicates that controlled dosing strategies could be employed to optimize therapeutic outcomes while minimizing adverse effects. The results demonstrated that the synthesized nanoparticles effectively inhibit cancer cell growth while sparing normal cells, suggesting their promise as a targeted nanomedicine approach.

**Fig. 6:**
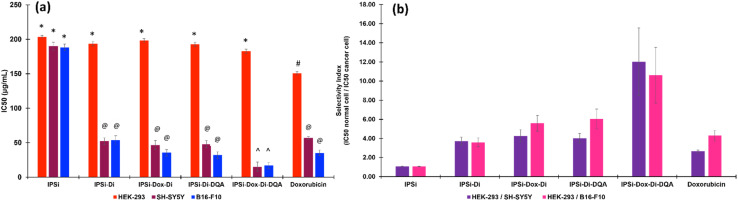
(A) IC_50_ values of the nanoparticles obtained from the normal cell human embryonic kidney HEK-293 and the cancer cells human neuroblastoma SH-SY5Y and murine melanoma B16-F10. The legends (*, @, #, ^) indicate significant differences at *p* > 0.05. (B) Selectivity indexes of the nanoparticles based on the ratio of the IC_50_ of the normal cell/cancer cell.

Based on the half-maximal concentrations (IC_50_) (Fig. 6a), the nanoparticle IPSi-Dox-Di-DQA showed the most cytotoxic activity against the two cancer cell lines with 15.24 (±6.54) μg mL^−1^ in SH-SY5Y and 17.23 (±4.46) μg mL^−1^ in B16-F10. These values are of significant cytotoxicity to the cancer cell lines based on the standards set by the U.S. National Cancer Institute.^52^ These IC_50_ values are also significantly different (*p* > 0.05) from the IC_50_ of the positive control doxorubicin. The IC_50_ values of IPSi-Di (52.25 μg mL^−1^ for SY-SY5Y and 54.02 μg mL^−1^ for B16-F10), IPSi-Dox-Di (46.64 μg mL^−1^ for SH-SY5Y and 35.57 μg mL^−1^ for B16-F10), and IPSi-Di-DQA (47.98 μg mL^−1^ for SH-SY5Y and 32.03 μg mL^−1^ for B16-F10) were statistically comparable (*p* < 0.05) with doxorubicin (56.87 μg mL^−1^ for SH-SY5Y and 35.21 μg mL^−1^ for B16-F10). The IC_50_ (180–205 μg mL^−1^) of the nanoparticles including the nanocarrier IPSi proved to be non-toxic against the HEK-293 normal cells.

The selectivity index (SI), as shown in Fig. 6b, is the ratio of the IC_50_ of the normal cell against the cancer cell. It is used to evaluate how effectively a drug inhibits the growth of cancer cells while sparing the normal cells.^53^ A high SI value (>2) may suggest a more effective inhibition of the growth of cancer cells than the normal cells. Hence, the SI is an excellent guide in determining drugs for further drug development. The nanoparticles, IPSi-Di, IPSi-Dox-Di, IPSI-Di-DQA, and IPSi-Dox-Di-DQA, and the positive control doxorubicin showed selective cytotoxicity (SI > 3) toward the cancerous cells as opposed to the normal cell. However, IPSi-Dox-Di-DQA gave the highest SI ranging from 10.6–12.0.

The SI analysis further supports the selective cytotoxicity of the nanoparticles toward cancer cells while sparing normal cells. The high SI values (>3) indicate their potential as effective anticancer agents with minimal toxicity to healthy tissues. Notably, IPSi-Dox-Di-DQA demonstrated the highest SI values highlighting its superior selectivity and efficacy. These findings emphasize the potential of IPSi-Dox-Di-DQA for further preclinical development as a promising candidate for targeted cancer therapy.

**Fig. 7:**
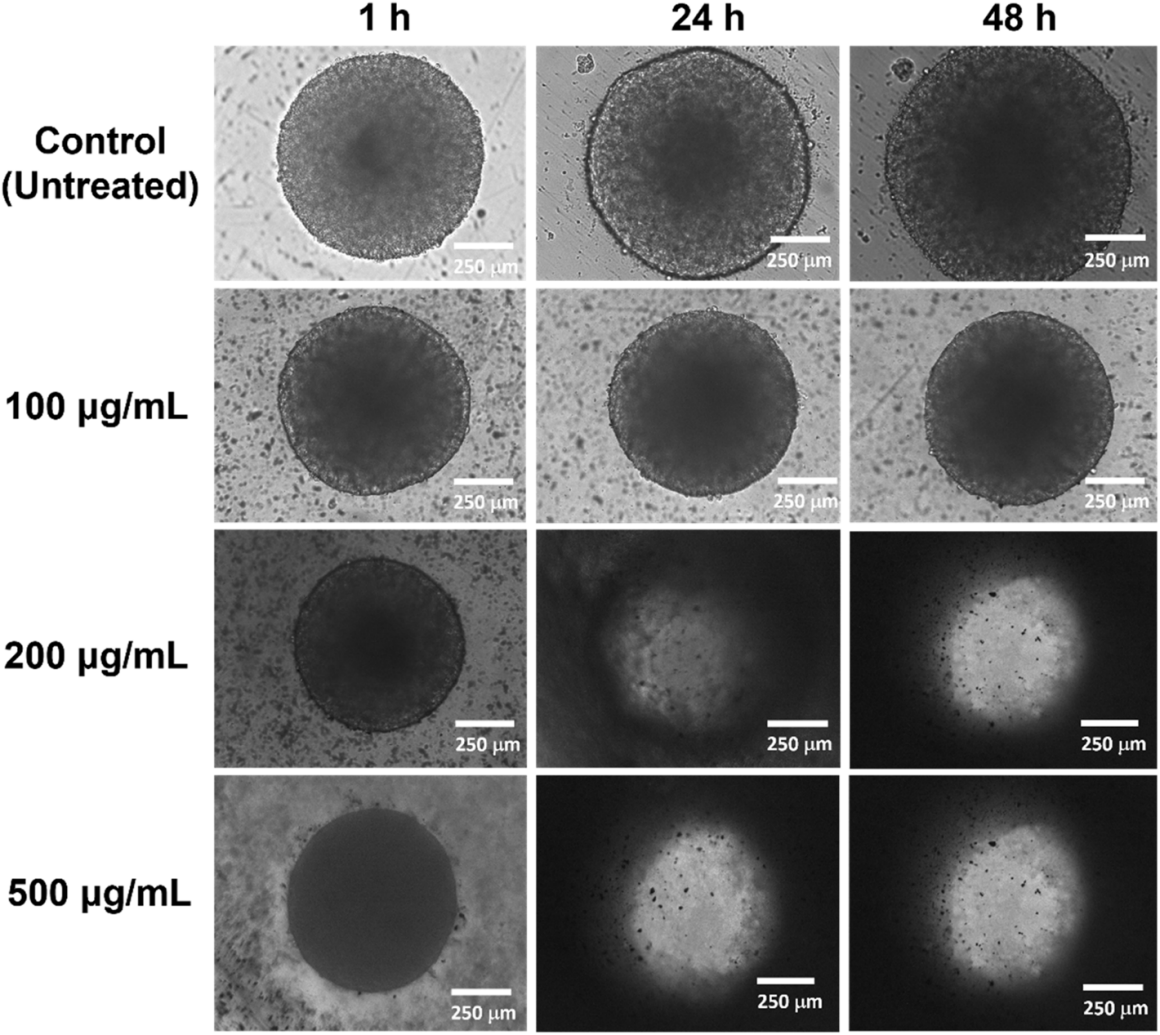
Representative images of the 3D cytotoxicity of IPSi-Dox-Di-DQA in (A) embryonic kidney HEK-293 normal cell. Cell morphology was monitored at 10× magnification after 1 h, 24 h, and 48 h of treatment.

**Fig. 8:**
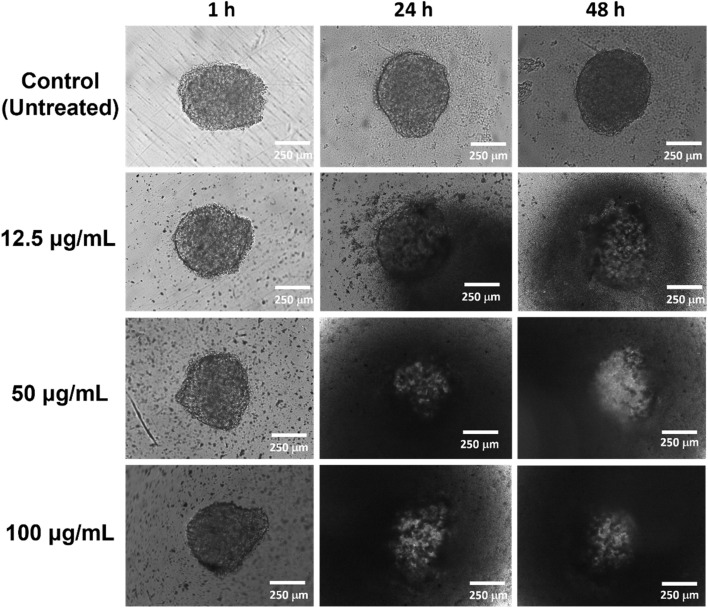
Representative images of the 3D cytotoxicity of IPSi-Dox-Di-DQA in neuroblastoma SH-SY5Y cancer cell. Cell morphology was monitored at 10× magnification after 1 h, 24 h, and 48 h of treatment.

To mimic the complexity of the normal and cancer cells *in vivo*, we also investigated the 3D cell viability of IPSi-Dox-Di-DQA using the HEK-293 (Fig. 7) and SH-SY5Y (Fig. 8) cells. Spheroidal tumor cells were visible after 5 days of incubation. A 3D cell viability of tumor cells is considered a more stringent model in an *in vitro* drug screening as 3D cell cultures acquire several *in vivo* characteristics of cancer cells.^54^*In vitro* 3D cell assays also take advantage of apprising information between 2D *in vitro* cell cultures and animal models and have been recommended as additional support to the conventional 2D cell assays before animal model studies.^54^ Based on the data of the IC_50_, 100, 200, and 500 μg mL^−1^ were used for HEK-293, while SH-SY5Y cells were treated with 12.5, 50, and 100 μg mL^−1^ concentrations. Cells were monitored after 1 h, 24 h, and 48 h of treatment. Untreated cells remained intact and continuously aggregating as observed by an increase in the diameter. The observed disintegration of spheroids upon IPSi-Dox-Di-DQA treatment suggests significant cytotoxic effects, which may be attributed to the enhanced drug penetration and retention within the 3D tumor-like structures. The progressive loss of spheroidal integrity aligns with previous reports^54,55^ indicating that nanoparticles can effectively disrupt tumor cell aggregates by inducing apoptosis and inhibiting cell proliferation. In the presence of a highly cytotoxic substance, spheroidal cells may undergo various changes in cellular morphology including cell shrinkage, loss of cell nucleation, and membrane blebbing.^55^ The high concentration of nanoparticles used in the HEK-293 cells may also further explain its observed 3D cellular cytotoxicity. Furthermore, the differential sensitivity between HEK-293 and SH-SY5Y cells highlights the importance of cell-type specificity in nanoparticle-mediated drug delivery, emphasizing the need for optimized dosing strategies in future therapeutic applications.

In summary, the synthesized nanoparticle IPSi-Dox-Di-DQA has shown great promise as a potential anticancer agent *in vitro*. However, it is important to acknowledge that the *in vitro* study only provides a preliminary anticancer assessment. To fully realize its potential as an effective anticancer agent, a comprehensive study is essential to address issues concerning its *in vivo* performance, pharmacokinetics, and safety profiles such genotoxicity and immunotoxicity. Notwithstanding this need for further investigation, the *in vitro* findings undeniably point to the considerable promise of IPSi-Dox-Di-DQA as an anticancer agent, underscoring the importance of continued research and development in finding therapy against cancer.

## 4 Conclusion

In conclusion, this study demonstrated the development of multifunctional mesoporous silicon nanoparticles for targeted, NIR-activated drug release and selective cancer cell cytotoxicity. The incorporation of dieckol and dequalinium enhanced chemo-photothermal sensitivity and controlled drug release. The IPSi-Dox-Di-DQA nanoparticles exhibited significant cytotoxic effects in cancer cells while sparing normal cells, as confirmed by 3D cell viability assessments. These findings highlight their potential for precision oncology, warranting further experiments including further elucidation of cytotoxicity mechanism, *in vivo*, immunotoxicity, and genotoxicity experiments to advance targeted cancer therapy.

## Consent for publication

All the authors agreed to publish their work in the present form.

## Data availability

Data for this study is available from the authors on request.

## Author contributions

Conceptualisation and methodology: Vy Anh Tran, Seong Soo A. An, Sang-Wha Lee, Mario A. Tan. Formal analysis and investigation: Vy Anh Tran, Nguyen Huy Hung, Thu Thao Thi Vo, Seong Soo A. An, Sang-Wha Lee, Hun Jeong, Mario A. Tan. Writing-original draft preparation: Vy Anh Tran, Thu Thao Thi Vo, Hun Jeong, Mario A. Tan. Writing—review and editing: Vy Anh Tran, Mario A. Tan. Funding acquisition: Vy Anh Tran Visualization: Vy Anh Tran, Nguyen Huy Hung, Thu Thao Thi Vo, Seong Soo A. An, Sang-Wha Lee, Mario A. Tan. Supervision: Vy Anh Tran, Mario A. Tan.

## Conflicts of interest

The authors declare that they have no known competing financial interests or personal relationships that could have appeared to influence the work reported in this paper.
